# Distribution of urinary N‐acetyl‐beta‐D‐glucosaminidase and the establishment of reference intervals in healthy adults

**DOI:** 10.1002/jcla.23748

**Published:** 2021-03-11

**Authors:** Qian Liu, Ruyuan Zong, Huan Li, Xiaoxiao Yin, Mei Fu, Li Yao, Jin Sun, Fumeng Yang

**Affiliations:** ^1^ Department of Laboratory Medicine The Second People's Hospital of Lianyungang Lianyungang China

**Keywords:** healthy adults, reference interval, urinary N‐acetyl‐beta‐D‐glucosaminidase

## Abstract

**Background:**

Urinary N‐acetyl‐beta‐D‐glucosaminidase (NAG) plays an important role in the early diagnosis and progression of diseases related to renal tubular injury. We detected the urinary NAG concentration, assessed the preliminary statistics of its distribution, and established reference intervals for healthy adults in China using the rate method.

**Methods:**

A total of 1,095 reference individuals (aged 20 to 79 years) met the requirements for inclusion in this study. Urinary NAG concentrations were detected using an AU5800 automatic biochemical analyzer with its matched reagents. The Kolmogorov‐Smirnov test was used to analyze the normality of the data. According to the guidelines of C28‐A3 and WS/T 402‐2012, the reference intervals of urinary NAG were established using the nonparametric percentile method (unilateral 95th percentile).

**Results:**

The urinary NAG data showed a non‐normal distribution. The distribution of urinary NAG was significantly different by sex and age. Therefore, the reference intervals of urinary NAG were established using the rate method: males (aged 20–59 years) <19.4 U/L (90% CI: 18.0–20.3 U/L); males (aged 60–79 years) <22.3 U/L (90% CI: 20.2–22.6 U/L); females (aged 20–59 years) <15.7 U/L (90% CI: 15.2–16.5 U/L); and females (aged 60–79 years) <21.4 U/L (90% CI: 20.3–22.3 U/L).

**Conclusions:**

We established preliminary reference intervals of urinary NAG for healthy adults in China to provide guidance for health screening, auxiliary diagnosis, and treatment monitoring of renal tubule‐related diseases.

## Graphical abstract

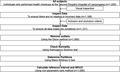

## 1 INTRODUCTION

N‐acetyl‐beta‐D‐glucosaminidase (NAG) is a high‐molecular‐weight lysosomal enzyme that is primarily expressed in proximal tubular epithelial cells.^1^, ^2^ The concentration of NAG in urine is very low under normal circumstances, but its secretion and urinary concentration increase significantly when renal tubular dysfunction leads to renal tubular epithelial cell damage.^3^, ^4^ Recent studies reported that urinary NAG is a sensitive biological indicator that plays an important role in the early diagnosis and progression of diseases related to renal tubular injury.^5^, ^6^, ^7^

Reference intervals are important components of laboratory indicators and provide an important basis for evaluating whether the body is functioning normally. If the reference interval is cited or established improperly, interference or incorrect interventions may occur in the clinic.^8^, ^9^ There are few reports of the establishment of reference intervals for urinary NAG.^10^, ^11^ The reference interval of urinary NAG was primarily derived from the instructions provided by reagent manufacturers, and its suitability requires further verification. Therefore, it is very important for laboratories to establish an appropriate reference interval to realize the full potential of the clinical application of urinary NAG. Therefore, we detected the concentration of urinary NAG, assessed the preliminary statistics of its distribution, and established reference intervals for healthy adults in China based on the rate method.

## 2 MATERIALS AND METHODS

### 2.1 Study subjects

**FIGURE 1:**
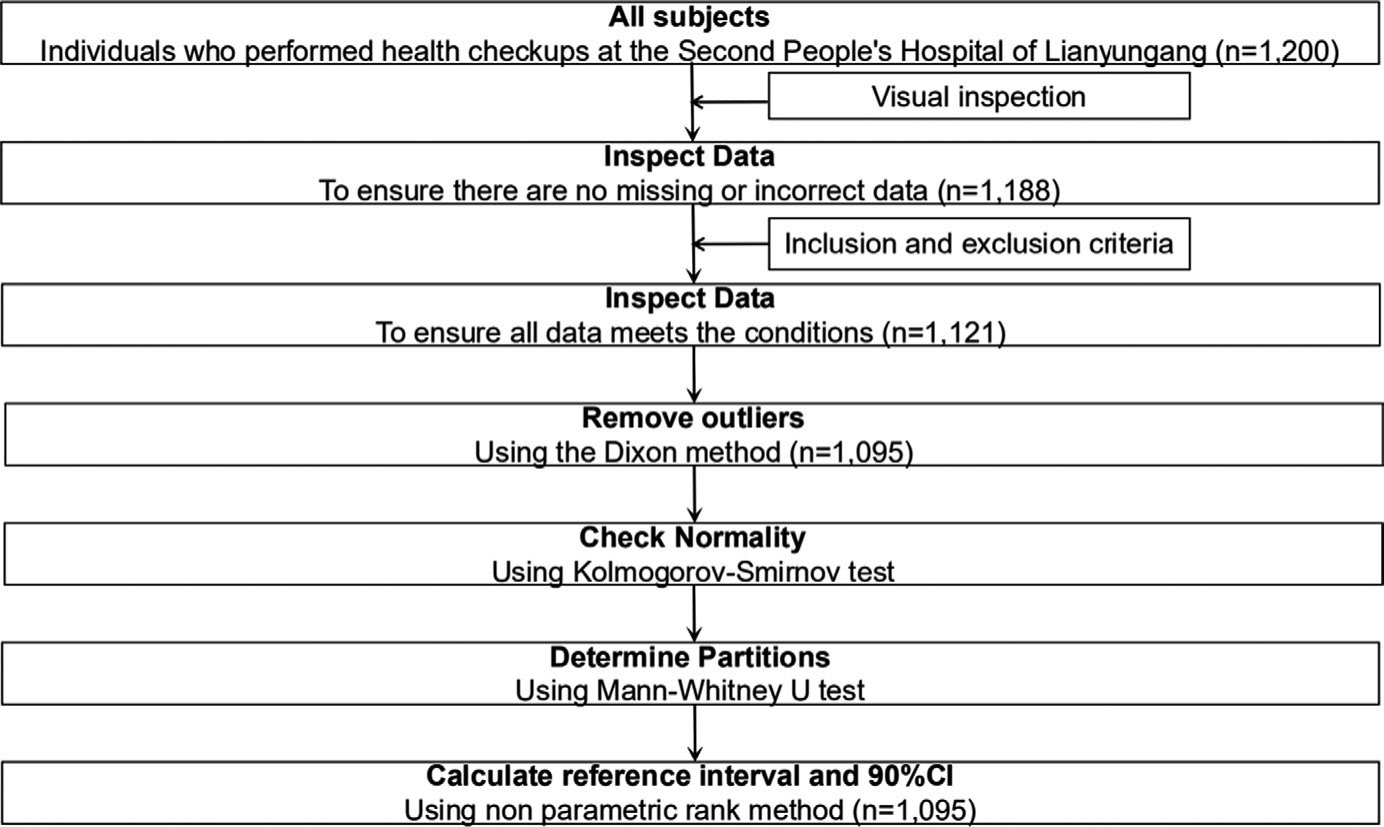
Establishing reference intervals of urinary NAG on the basis of C28‐A3

According to a complete randomness method, 1200 subjects who completed a physical examination at the Physical Examination Center of the Second People's Hospital of Lianyungang from September 2020 to October 2020 were selected as participants in this study. Based on the inclusion and exclusion criteria, a total of 1095 reference individuals (548 males and 547 females; aged 20–79 years old) conformed to the requirements of this study. Subjects were divided into a male group (aged 20–79 years old) and female group (aged 20–79 years old) based on the guidelines of the Clinical and Laboratory Standards Institute (CLSI) C28‐A3 ^12^ and WS/T 402‐2012.^13^ Participants were further divided according to age into male (aged 20–59 years old), male (aged 60–79 years old), female (aged 20–59 years old), and female (aged 60–79 years old) groups. The reference interval was determined independently or combined according to whether the distribution of urinary NAG was significantly different between the groups. The detailed study participant screening procedures are shown in Figure 1.

Participants in this study met the following inclusion criteria: (1) aged 20 to 79 years, body mass index (BMI) of 18.5–28.0 kg/m^2^, and blood pressure less than 139/89 mmHg; (2) serum urea (urea), creatinine (Cr), uric acid (UA), total cholesterol (TC), triglyceride (TG), high‐density lipoprotein cholesterol (HDL‐C), low‐density lipoprotein cholesterol (LDL‐C), fasting glucose (GLU), and other biochemical indicators in the normal reference interval; (3) normal urinary routine indicators; and (4) negative for all infectious disease indicators, such as hepatitis B, hepatitis C, and treponema pallidum (Table 1). Participants were excluded if they had (1) various acute traumas, acute or chronic infections; (2) digestive system diseases, kidney diseases, or various autoimmune diseases; (3) endocrine disorders, such as Cushing syndrome, hyperthyroidism, or hypothyroidism; (4) received surgical treatment in the past six months; (5) received medication within the past month; or (6) irregular work and rest schedules, overeating, alcoholism, or smoking. The Medical Ethics Committee of the Second People's Hospital of Lianyungang approved this study, and all participants signed informed consent forms.

**TABLE 1:** Conventional characteristics of reference individuals

Indicators	All (*n* = 1095)	Male (*n* = 548)	Female (*n* = 547)	Reference interval
Age (years)	50 (36, 62)	48 (37, 60)	52 (33, 63)	–
BMI (kg/m^2^)	22.8 ± 2.1	23.0 ± 1.8	22.6 ± 2.3	18.5–28.0
GLU (mmol/L)	4.99 ± 0.47	5.11 ± 0.53	4.87 ± 0.42	3.89–6.11
Urea (mmol/L)	5.6 ± 0.6	5.7 ± 0.5	5.4 ± 0.7	3.1–8.0 (male, aged 20–59 years) 3.6–9.5 (male, aged 60–79 years) 2.6–7.5 (female, aged 20–59 years) 3.1–8.8 (female, aged 60–79 years)
Crea (µmol/L)	56 ± 16	59 ± 14	52 ± 18	57–97 (male, aged 20–59 years) 57–111 (male, aged 60–79 years) 41–73 (female, aged 20–59 years) 41–81 (female, aged 60–79 years)
UA (µmol/L)	292 ± 36	309 ± 42	276 ± 38	208–428 (male) 155–357 (female)
TC (mmol/L)	4.38 ± 0.36	4.45 ± 0.28	4.30 ± 0.32	<5.17
TG (mmol/L)	1.04 ± 0.22	1.05 ± 0.23	1.03 ± 0.20	<1.70
HDL‐C (mmol/L)	1.02 ± 0.21	0.98 ± 0.19	1.05 ± 0.23	0.91–1.66 (male) 0.91–1.74 (female)
LDL‐C (mmol/L)	2.64 ± 0.28	2.68 ± 0.27	2.59 ± 0.29	<3.37

Abbreviations: BMI, body mass index; Crea, creatinine; GLU, glucose; HDL‐C, high‐density lipoprotein cholesterol; LDL‐C, low‐density lipoprotein cholesterol**;**TC, total cholesterol; TG, triglyceride; UA, uric acid; Urea, urea.

### 2.2 Specimen collection

All subjects were instructed to maintain a normal diet for 3 days before the physical examination, and 5 mL of fasting venous blood was collected. The specimens were centrifuged at 3000 rpm for 10 min and detected within 6 h. Two 10‐ml tubes of fresh morning urine were also collected. Measurements of urinary routine indicators and urinary NAG were performed within 2 and 6 h, respectively.

### 2.3 Instruments and reagents

Based on the reagent instructions, the concentrations of serum urea, Cr, UA, GLU, TC, TG, HDL‐C, and LDL‐C were analyzed by the detection platform of the AU5800 automatic biochemical analyzer (Beckman Coulter) with the corresponding reagents (urea kit lot: AUZ7255; Cr kit lot: 2475; UA kit lot: AUZ7211; GLU kit lot: AUZ7246; TC kit lot: AUZ7179; TG kit lot: AUZ7125; HDL‐C kit lot: AUZ7437; LDL‐C kit lot: AUZ7687; Beckman Coulter). The urinary NAG level was also detected using an AU5800 automatic biochemical analyzer (Beckman Coulter., Ltd.) with matching reagents (NAG kit lot: 200902; Zhongyuan Biotechnology). Urinary routine indicators were detected using an AVE‐772 automatic urine analyzer (AVE) with matching reagents. All instruments were effectively maintained and calibrated before sample detection, and internal quality control was performed. The results of an external quality assessment were qualified.

### 2.4 Statistical analyses

#### 2.4.1 Outlier test

The Dixon method was applied to the outlier test, and all results were sorted by size (from small to large) to calculate the range (R). The difference (D) between the maximum (or minimum) result and its adjacent result was also calculated. If D/R was greater than or equal to 1/3, then the maximum (or minimum) result was eliminated as an outlier. The above steps were repeated until all outliers were removed.^12^

#### 2.4.2 Data analysis

The urinary NAG data were analyzed using SPSS Statistics, version 19 (IBM). The Kolmogorov‐Smirnov test was used to analyze normality. Nonparametric data are expressed as the median (M) and interquartile range (IQR). The Mann–Whitney *U* test was used to compare the distribution of data between the two groups (sex or age), and determine groups or combinations based on the statistical results to formulate reference intervals for urinary NAG. The nonparametric percentile method (95% single‐sided, 90% confidence interval for the upper limit of the reference interval) was used to establish reference intervals for urinary NAG. Differences were statistically significant at *p* < 0.05.

## 3 RESULTS

**FIGURE 2:**
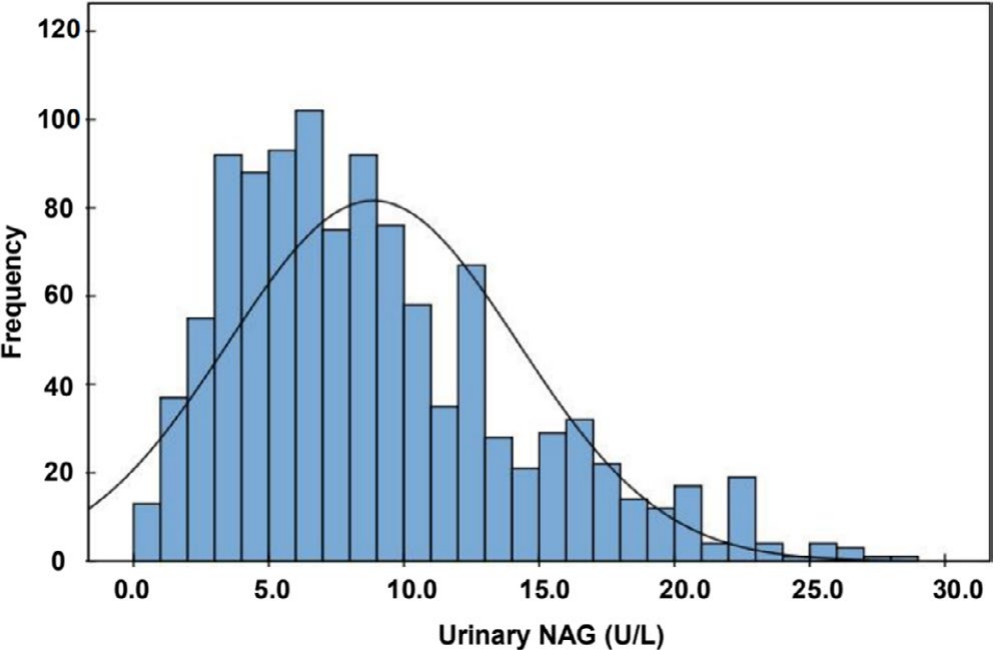
Distribution histogram of urinary NAG levels

According to the inclusion and exclusion criteria, 1095 subjects were ultimately included in this study, and their conventional indicators are shown in Table 1. The Kolmogorov‐Smirnov test showed a non‐normal distribution for urinary NAG levels (*p* < 0.05; Figure 2). The urinary NAG concentrations in males were significantly higher than females (*Z* = −7.269, *p* = 0.000; Table 2). Significant age‐related differences in the levels of urinary NAG were found and showed that the urinary NAG concentrations increased with age (males: *Z* = −3.025, *p* = 0.002; females: *Z* = −3.112, *p* = 0.002, Table 3).

**TABLE 2:** Distribution of urinary NAG by sex

Sex	*n*	M (IQR)	Upper limit	Mann–Whitney *U* test
Male	548	8.6 (6.0–12.6)	20.3 (90% CI: 19.8–22.2)	*Z* = −7.269 *p* = 0.000
Female	547	6.6 (3.7–10.7)	17.4 (90% CI: 16.5–20.5)	

Note

The unit of urinary NAG is U/L.

Abbreviations: CI, confidence interval; IQR, interquartile range; M, median.

**TABLE 3:** Distribution of urinary NAG by age

Age group	*n*	M (IQR)	Upper limit	Mann–Whitney *U* test
Male (aged 20–59 years)	278	8.1 (5.8–11.4)	19.4 (90% CI: 18.0–20.3)	*Z* = −3.025 *p* = 0.002
Male (aged 60–79 years)	270	9.6 (6.1–13.7)	22.3 (90% CI: 20.2–22.6)	
Female (aged 20–59 years)	284	6.1 (3.6–9.5)	15.7 (90% CI: 15.2–16.5)	*Z* = −3.112 *p* = 0.002
Female (aged 60–79 years)	263	7.7 (3.8–12.3)	21.4 (90% CI: 20.3–22.3)	

Note

The unit of urinary NAG is U/L.

Abbreviations: CI, confidence interval; IQR, interquartile range; M, median.

In accordance with the nonparametric methods recommended by the CLSI C28‐A3 and WS/T 402‐2012 and combined with the clinical value of urinary NAG, the upper reference limit is very important, and it was set at the unilateral 95th percentile. Therefore, the following reference intervals of urinary NAG were determined using the rate method: males (20–59 years old) <19.4 U/L (90% confidence interval: 18.0–20.3 U/L); males (60–79 years old) <22.3 U/L (90% confidence interval: 20.2–22.6 U/L); females (20–59 years old) <15.7 U/L (90% confidence interval: 15.2–16.5 U/L); and females (60–79 years old) <21.4 U/L (90% confidence interval: 20.3–22.3 U/L).

## 4 DISCUSSION

Since 2012, the National Health Commission of the People's Republic of China has established a series of biological reference intervals for laboratory assays based on multicenter studies, and these series of intervals provide an important basis for the correct citation of the reference intervals for assays.^14^, ^15^, ^16^ However, several factors, such as population characteristics, regional characteristics, and living habits, affect the reference intervals, and it is particularly important for each laboratory to determine reference intervals that are suitable for populations in different regions. The correctness of the reference interval directly affects the diagnosis of clinical diseases and the choice of treatment measures.^17^, ^18^

Based on the characteristics of the population in China, 1095 healthy adults who met the inclusion and exclusion criteria were enrolled in this study.

Our study indicated that the urinary NAG concentrations in males were significantly higher than females. We also performed statistical analysis based on age group. The results showed that there was a significant difference in urinary NAG concentrations between healthy adults according to age. The levels of urinary NAG tended to increase with age. Cheng et al^19^ indicated that the levels of urinary NAG were not significantly different between males and females, and the difference between age groups (aged 20–59 and aged 60–79) was not statistically significant. However, their study also indicated that the concentration of urinary NAG in people above 70 years old was significantly higher than people under 70 years old. These research results are different from the results of our study. The following possible reasons may explain this difference: (1) The source of reference individuals were different, which may have led to differences in population characteristics, eating habits, etc.; (2) the number of subjects was obviously different, and both studies have the possibility of sampling error; and (3) the different detection systems may lead to differences.

The levels of urinary NAG do not have clinical value at the lower limit of the reference interval and present a non‐normal distribution. Therefore, the unilateral 95th percentile (P_95_) was used to establish the reference intervals for urinary NAG, which was based on the nonparametric method recommended by the CLSI C28‐A3 and WS/T 402‐2012 guidelines. The present study preliminarily established the following reference intervals of urinary NAG for healthy adults in China based on sex and age: males (20–59 years old) <19.4 U/L; males (60–79 years old) <22.3 U/L; females (20–59 years old) <15.7 U/L; and females (60–79 years old) <21.4 U/L. Qin et al^20^ recently reported that the reference interval of urinary NAG provided by the manufacturer of the kit was 0.3–12.0 U/L, which was significantly lower than the reference intervals of urinary NAG established in our study. The main reason for this difference may be related to different population characteristics. Omozee et al^21^ showed that the average concentration of urinary NAG in a healthy population was 21.8 U/L. The median urinary NAG levels for each group in our study were lower than 10 U/L. The results reported by Omozee et al^21^ were twice as high as the results of our study. Combined with the above results, our study further confirms that the reference interval has important associations with population and regional characteristics. Therefore, a laboratory's self‐built reference interval has important guiding significance for the clinical application of each assay.

However, there are three limitations in this study. First, this study was a single‐center study with a relatively limited number of reference individuals, and its overall representativeness is slightly low. Second, we primarily focused on healthy adults aged 20–79 years, and we lacked the ability to determine reference intervals for children, adolescents, and pregnant women. Therefore, this reference range was not applicable to these populations. Third, although the reference individuals included in this study met the requirements for establishing a reference interval, the number of cases was insufficient, and more individuals should be included to obtain a better overall representativeness.

In conclusion, we preliminarily established the reference intervals of urinary NAG for healthy adults in China using the rate method, which provides guidance for health screening, auxiliary diagnosis and treatment monitoring of renal tubule‐related diseases.

## CONFLICT OF INTEREST

The authors declare that they have no conflicts of interest associated with this work.

## AUTHORS’ CONTRIBUTIONS

QL and RYZ researched the literature and conceived the experiments. MF, LY and JS were involved in protocol development, ethical approval, patient recruitment and data analyses. QL, RYZ and FMY wrote the first draft of the article. All authors reviewed and edited the article and approved the final version of the article.

## Data Availability

Data available on request from the authors.
